# Supplementary material for: Data-driven fine-grained region discovery in the mouse brain with transformers
Source: Nat Commun. 2025 Oct 7;16:8536. doi: 10.1038/s41467-025-64259-4 (PMC12504640; doi:10.1038/s41467-025-64259-4)
Supplement: Supplementary file 1 — Supplementary Information [file 41467_2025_64259_MOESM1_ESM.pdf]

Supplementary Information

Supplementary Note 1: Effect of smoothing and analysis on striatal glial populations

When scaling up the number of regions past 500 in the Allen 1 dataset[1], we observed that almost all spatial clusters were spatially smooth except for a recurring pattern in the striatum. We plot (**Supplementary Figure 19a-b**) six sequential sections where we identified an irregular (which we define here as a broadly non-convex shape that does not form a relatively singular connected component) pattern of cells in the striatum and only the striatum (note the spatial uniformity of areas surrounding striatum in cortex and endopiriform area, nucleus accumbens, etc.). We identified cells in these areas and found they were mostly non-neuronal, with astrocytic types (such as 1163 **Astro-TE NN_3, Supplementary Figure 19**) forming a large proportion of cells.

We sought to understand whether these spatial clusters might be biologically relevant or somehow related to noise. A recent paper, Ollivier et al. (2024)[2] identified a novel population of *Crym*+ astrocytes in a similar spatial distribution as observed in our regions, specifically in a dorsoventral and lateromedial distribution (see **Supplementary Figure 19e,** reproduced with permission from Ollivier et al. (2024)[2]). As *Crym* was included in the MERFISH panel, we quantified *Crym* expression in astrocytes within these areas, finding that all but two of these spatially irregular domains had very high levels of *Crym* expression. Notably, the two groups with lower expression, spatial clusters 457 and 758, were the most dorsolateral and are distributed where *Crym*+ astrocytes were not observed in Ollivier et al. We reasoned that these spatial clusters may have biological relevance.

However, to simplify downstream analyses and conform with neuroanatomical conventions, we applied a simple smoothing operation (see **Methods**), which removed this spatial cluster in successive clustering operations. We used a very small smoothing window (12-micron sigma, or 40 micron full-width at half-maxima) and found the order of ranked methods and their relative performance changes were not significantly affected.

To clarify the effect of smoothing on subsequently discovered domains, see **Supplementary Figure 11**, which shows roughly the same sections as **Supplementary Figure 19** (containing the striatal non-contiguous domains), and also shows that in the unsmoothed embeddings, non-contiguous domains are limited to the striatum and only between sections 45 and 52. More anterior sections (past section 55) do not demonstrate any non-contiguous domains, and prior to section 43, there are also no non-contiguous domains. This can be seen at a larger scale in **Supplementary Figure 12** across the entire Allen 1 dataset.

Supplementary Note 2: Interpreting the CellTransformer objective as a measure of spatial dependence

One interpretation of the CellTransformer architecture is learning two representations of cellular gene expression. The first (the learned embedding for each cell type) is unconditional on the spatial neighborhood information. The second is one that is conditional on the spatial neighborhood information learned in the encoder portion of the network and parameterized as a residual update. This residual update can then conveniently be aggregated across layers and represented as a single update term on the unconditional representation to produce the final output. We interpret the increase in accuracy from neighborhood-conditional gene expression prediction as an index of spatial dependence. A trivial or poorly fit optimization solution would produce a small value of this index. A similar idea has been previously presented in a number of works, most recently the NCEM approach[3].

When analyzing the Allen 1 dataset, we observe increases in the predictive accuracy (mean 0.10 +/- 0.0701 in correlation, averaged across cell types) across the dataset. Moreover, there are a few cell types for which there is a decrease in predictive accuracy, indicating that our model has nontrivially learned the objective (**Supplementary Figure 20a**). Those that are poorly predicted are often only present in the dataset at very low abundances. Conditioned on cell types with more than 10^2-10^3 cells, accuracy has only a mild correlation with cellular density (**Supplementary Figure 20b**) or with the number of cells of a given type in the dataset. Immature neurons (IMN) are the class that benefits the most from conditional prediction, suggestive of their complex migratory dynamics[1]. Note that we compute accuracies at the subclass level (338 types). Increase in accuracy does correlate strongly with log-number of observations per cell type, (Pearson correlation of 0.71, **Supplementary Figure 20c**), however cells that do not see an increase in prediction accuracy are a very small proportion of the total number (6 out of 1201 supertypes, or 0.5%), and 5 out of 6 of these are only found with double-digit abundances across the brain (less than 0.1% in total across all 5 types).

Supplementary Figures

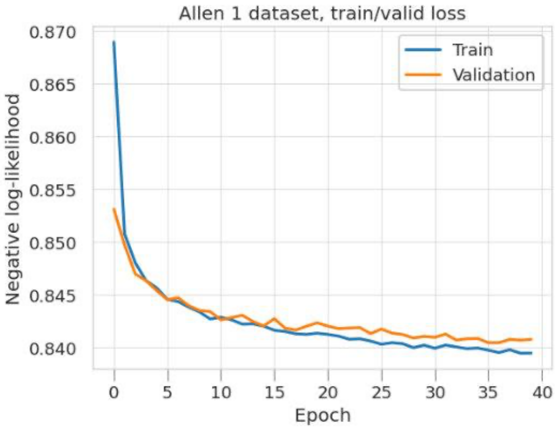

Supplementary Figure 1. Train and validation loss curves for CellTransformer trained on the Allen 1 dataset[1].

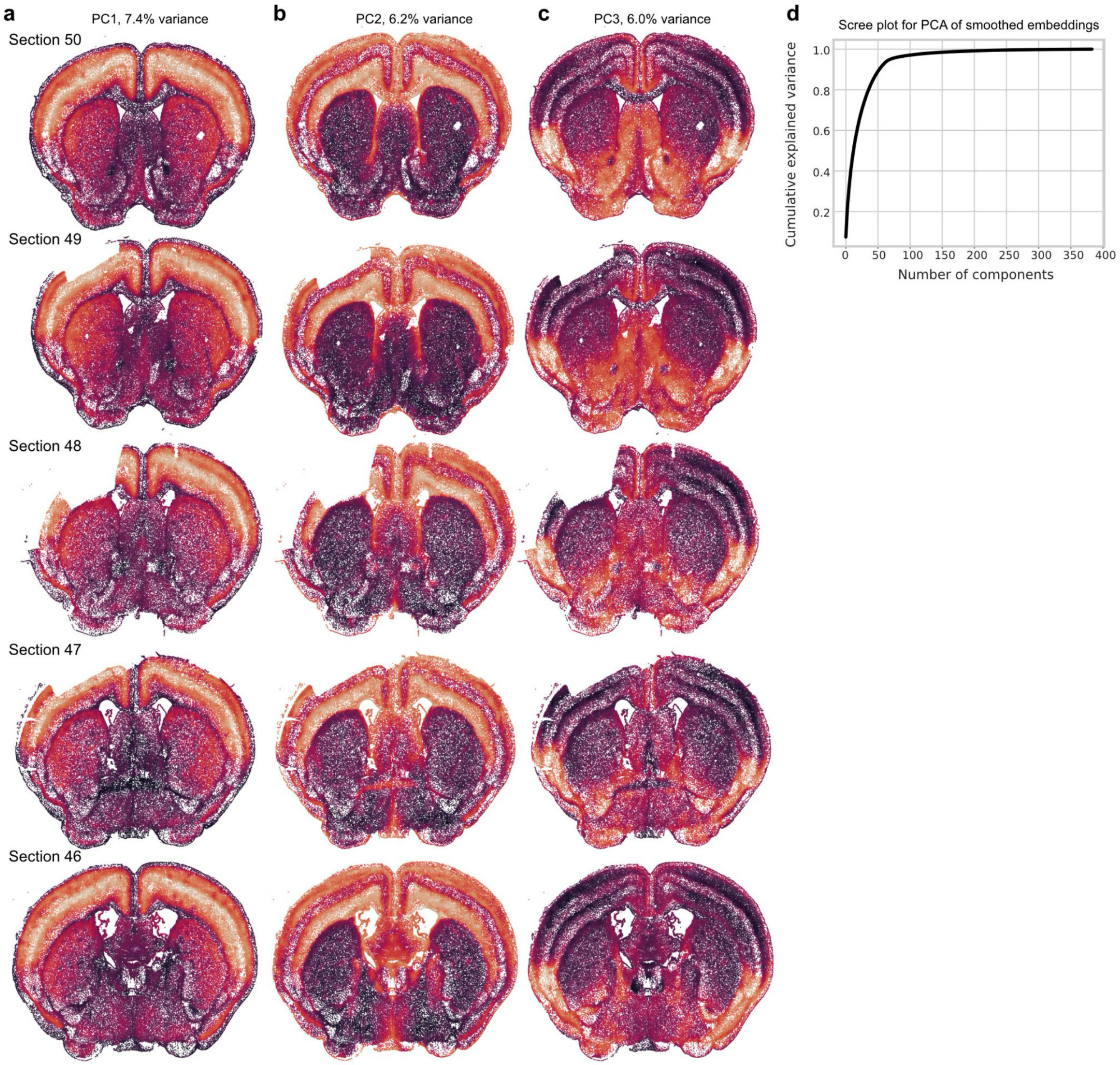

Supplementary Figure 2. Visualizations of leading principal components of CellTransformer embeddings across several tissue sections in the Allen 1 dataset[1]. (**a.-c.**) Principal components 1-3, in total constituting 19.5% of dataset-wide variance, across five sequential sections. (**d.**) Scree plot of variance explained by component.

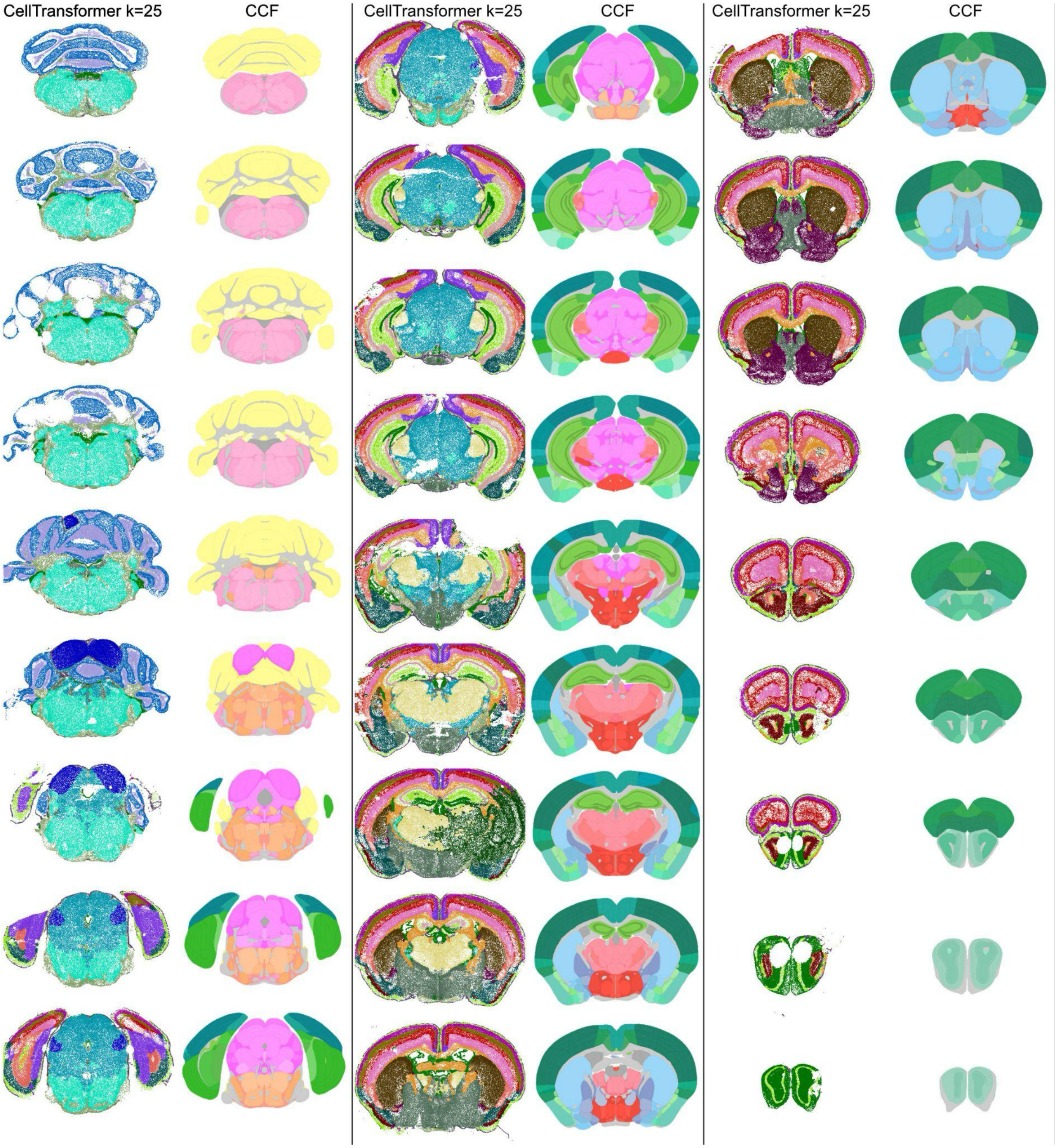

Supplementary Figure 3. CellTransformer spatial domains (left) and the corresponding CCF annotations (right) organized in 3 columns for roughly half of the sections in the Allen 1 dataset[1], approximately every other section. CellTransformer domains were calculated at *k*=25 clusters.

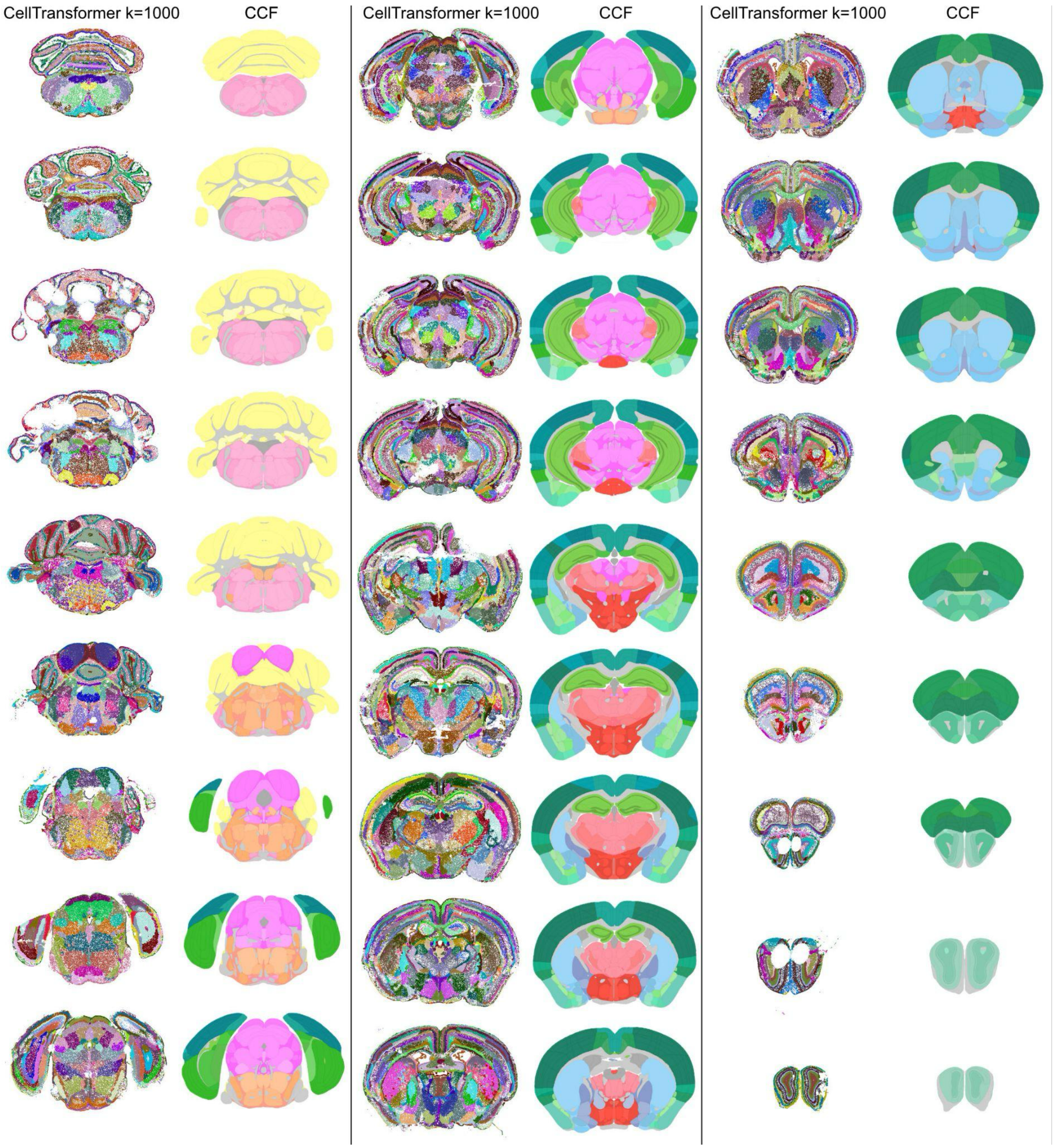

Supplementary Figure 4. CellTransformer spatial domains (left) and the corresponding CCF annotations (right) organized in 3 columns for roughly half of the sections in the Allen 1 dataset[1], approximately every other section. CellTransformer domains were calculated at *k*=1000 clusters.

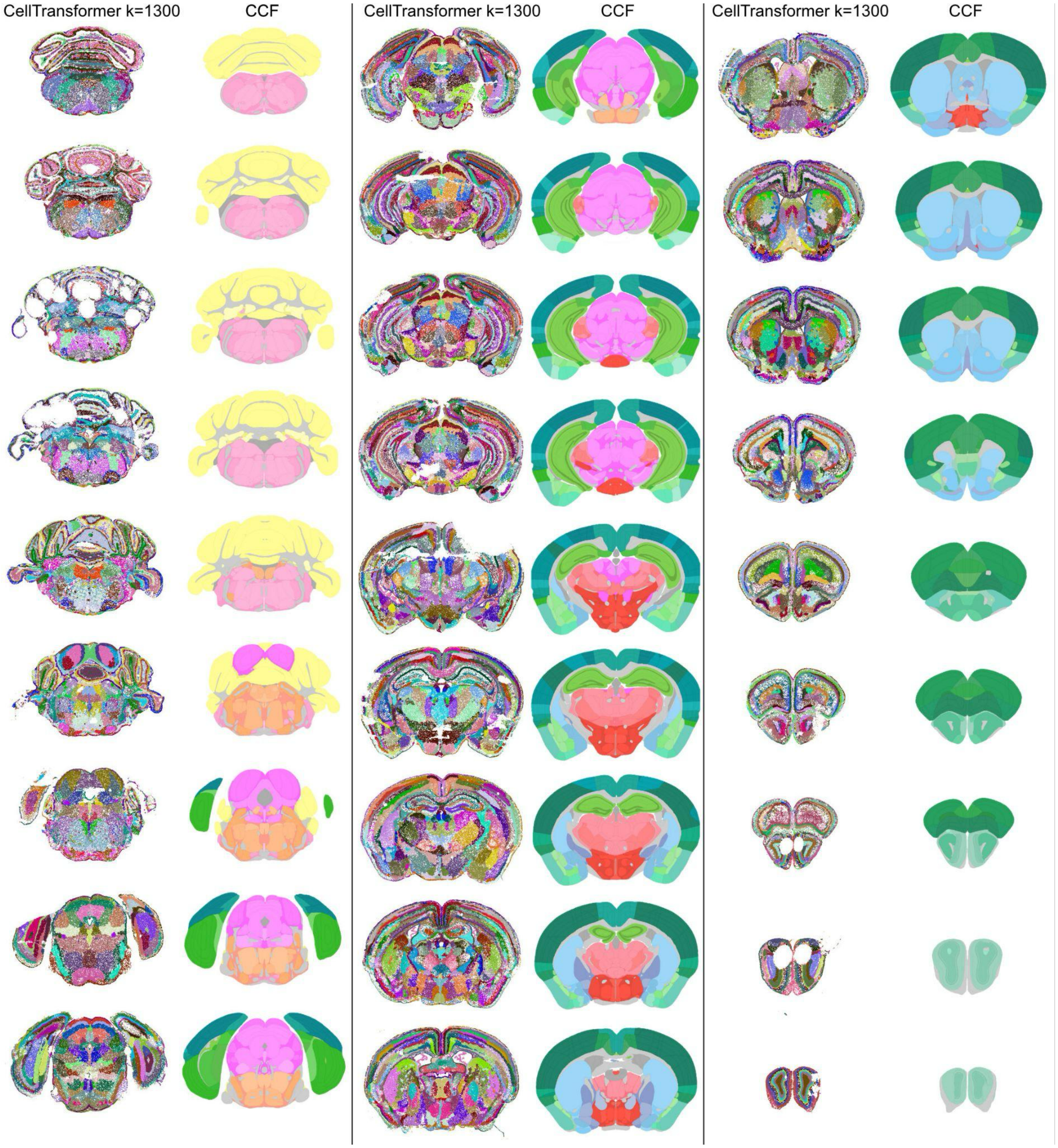

Supplementary Figure 5. CellTransformer spatial domains (left) and the corresponding CCF annotations (right) organized in 3 columns for roughly half of the sections in the Allen 1 dataset[1], approximately every other section. CellTransformer domains were calculated at *k*=1300 clusters.

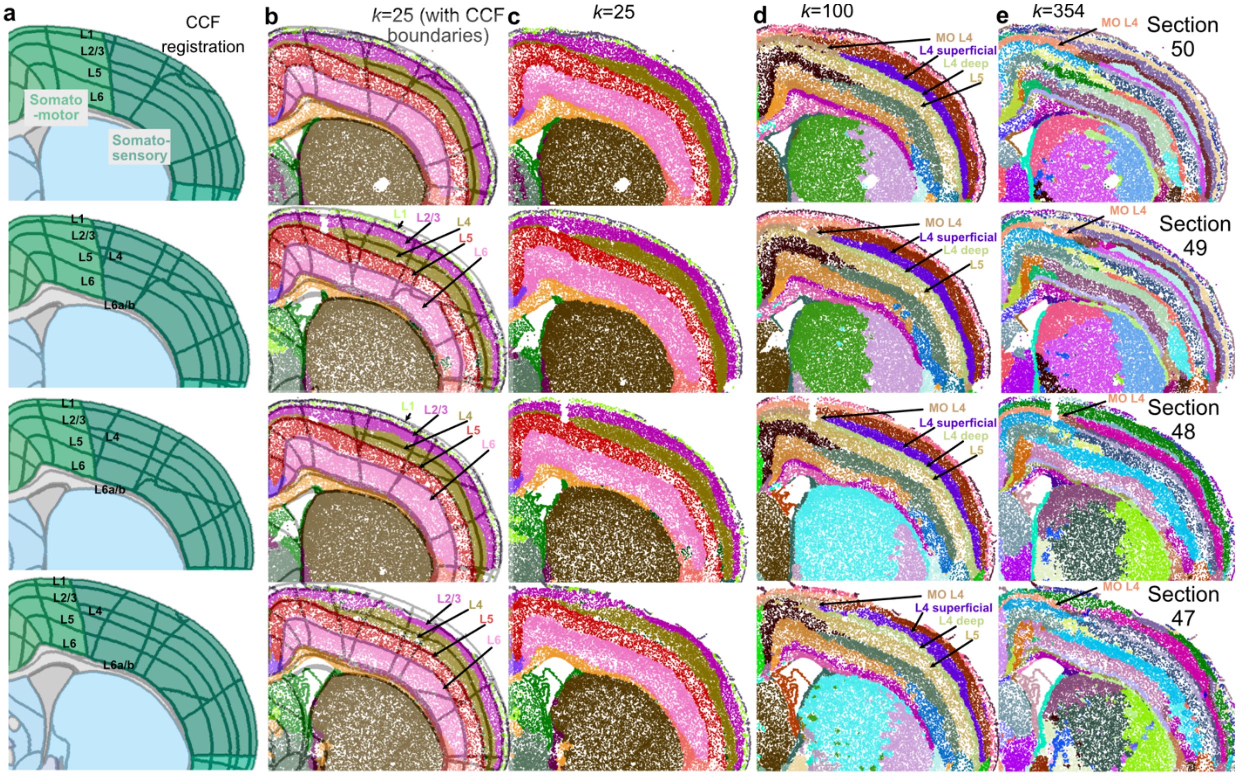

Supplementary Figure 6. Four sequential sections of the Allen 1 dataset[1] (200 μm sampling interval between sections), displayed with CellTransformer labels at varying resolution alongside CCF registration results. MO: motor cortex. (**a.**) CCF registration of four sequential sections is shown in **Figure 2**. The cortical layers are marked based on CCF annotations. (**b.**) *k*=25 spatial domains with CellTransformer shown with regional boundaries from CCF in light gray. Putative cortical layers are annotated, showing CellTransformer replicates known cortical layers. (**c.**) 25 domains shown without CCF annotations to facilitate visualization. (**d.**) Same sections now shown with 100 domains to help show the transition from coarse (25 domains) to fine (100 domains). Sublayers of cortex are identified, including layer 4 in the motor cortex, which transcriptomic studies have verified, but have been difficult to identify using histological approaches. (**e.**) 354 domain zoom-in on the same sections, showing consistency of layer 4 motor cortex detection as well as an anterior-posterior subdivision across motor and somatosensory cortical layers and clear distinction of cortical layers that lie within motor and somatosensory areas.

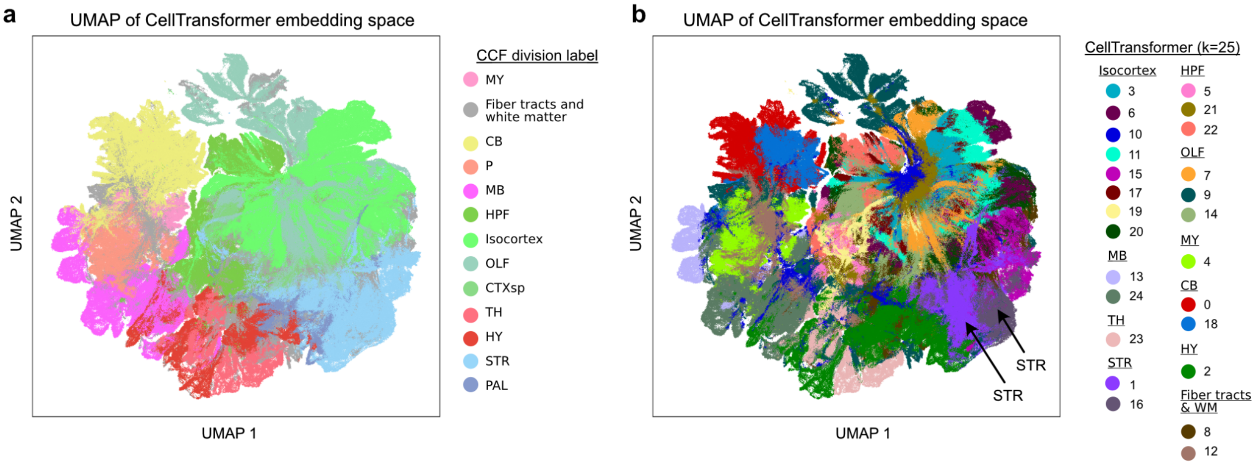

Supplementary Figure 7. UMAP of smoothed CellTransformer embeddings on the Allen 1 dataset[1]. (**a.**) UMAP colored by CCF division (25 total) annotation for each cell; the "fiber-tracts-unassigned', "mfbs", "cbf", "eps", "cm", "V4", "VL", "AQ", "scwm", and "V3" areas were collapsed into the "Fiber tracts and white matter" label to make the visualization simpler. (**b.**) UMAP colored by CellTransformer (*k*=25) domains. Domains are grouped by the CCF division with the greatest spatial overlap. Domains corresponding to striatum (domains 1 and 16) are highlighted with black arrows. Abbreviations: mfbs-medial forebrain bundle system; cbf-cerebellum related fiber tracts; cm-cranial nerves; eps-external plexiform layer; V3-third ventricle; V4-fourth ventricle; VL-lateral ventricle; scwm-superior colliculus commissure; AQ-cerebral aqueduct; MY-medulla; P-pons; MB-midbrain; HPF-hippocampal formation; OLF-olfactory bulb; TH-thalamus; CTXsp-cortical subplate; HY-hypothalamus; STR-striatum; PAL-pallidum.

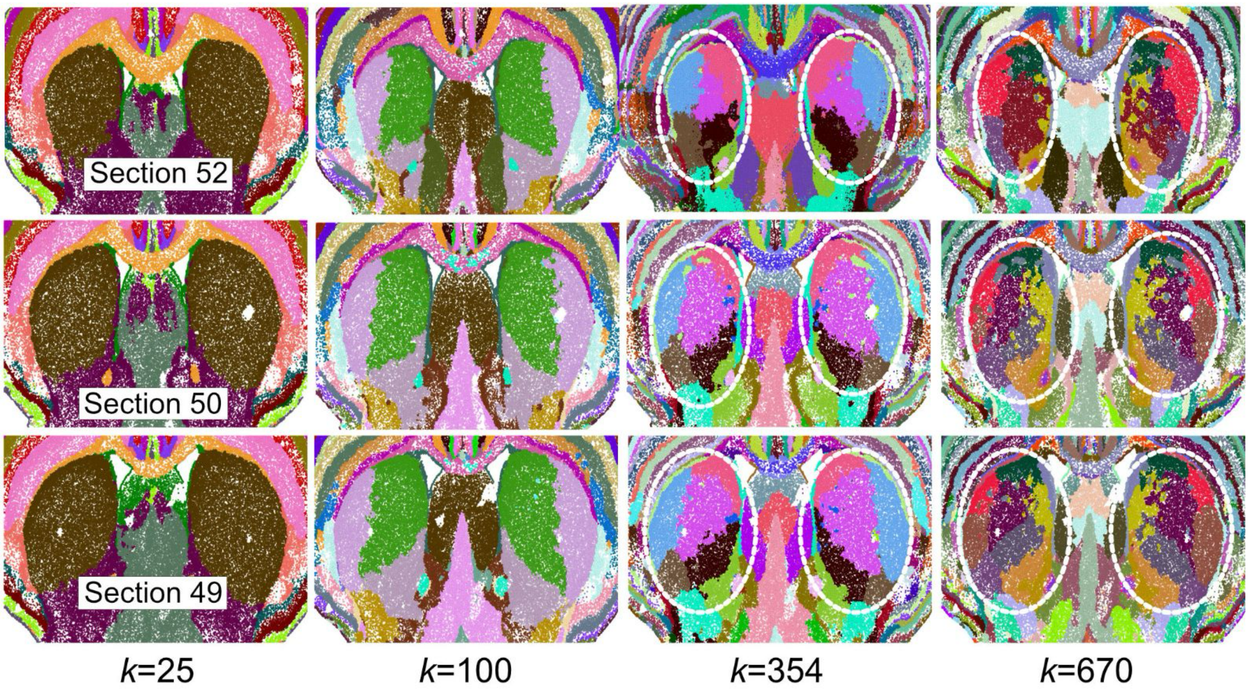

Supplementary Figure 8. CellTransformer domains identified in the Allen 1 dataset[1] at varied values of *k*, colored in three sequential sections (200 μm sampling interval between sections with consecutive numbers, top to bottom corresponds to rostral to caudal). Caudoputamen is roughly highlighted by dotted circles in *k*=354 and *k*=670 to assist visualization.

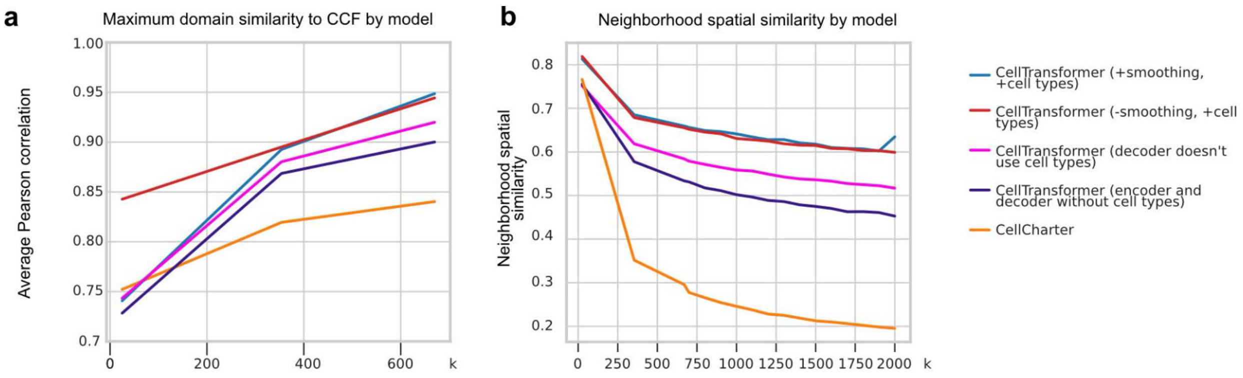

Supplementary Figure 9. Comparison of different CellTransformer variants (smoothed versus unsmoothed embeddings; models with and without cell type information in the transformer encoder and decoder) in the Allen 1 dataset[1]. (**a.**) Average max correlation of data-driven domains to CCF. (**b.**) Neighborhood spatial similarity, averaged across all cells by models.

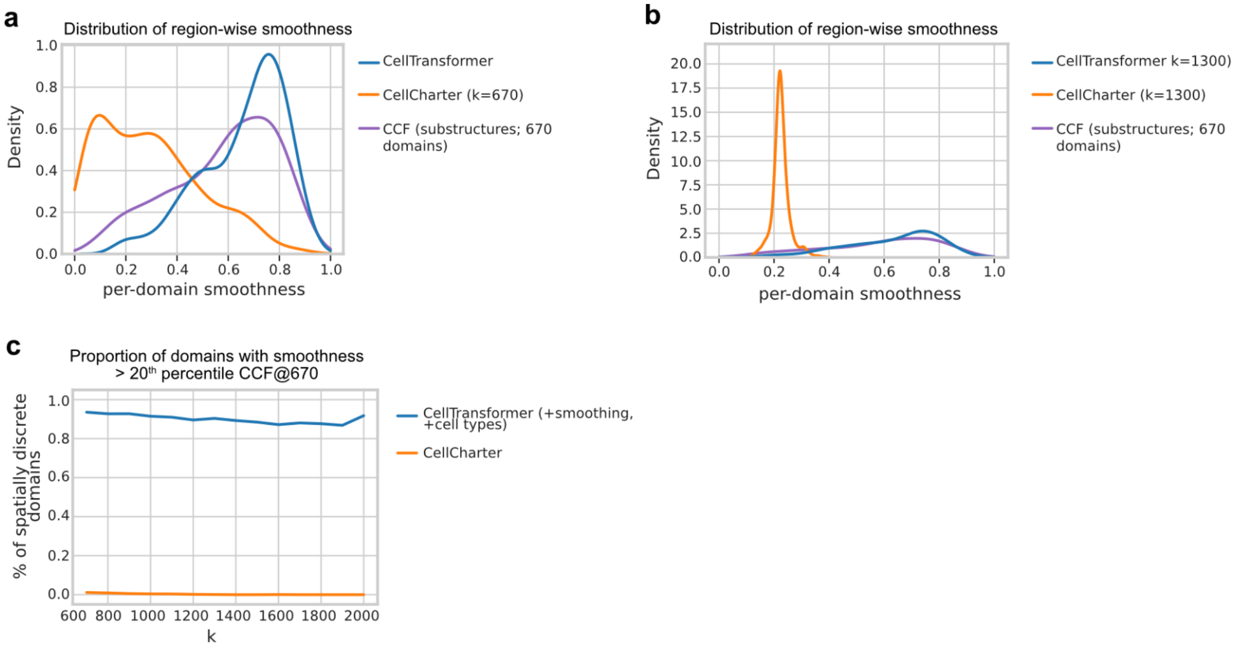

Supplementary Figure 10. Visualizations of the distribution of per-domain spatial smoothness comparing CCF and various data-driven spatial domains. All sections are from the ABC-WMB (Allen 1 dataset)[1]. (**a.**) Distribution of per-region spatial smoothness for 670 CCF domains compared with 670 CellCharter[4] and 670 CellTransformer domains (shown with smoothing). (**b.**) Distribution of per-region spatial smoothness for 670 CCF domains, compared with CellCharter and CellTransformer at 1300 domains. (**c.**) Proportion of discrete domains using a fixed cutoff at 20th percentile of per-region CCF smoothness values (0.381), applied to CellCharter and CellTransformer from 700 to 2,000 domains.

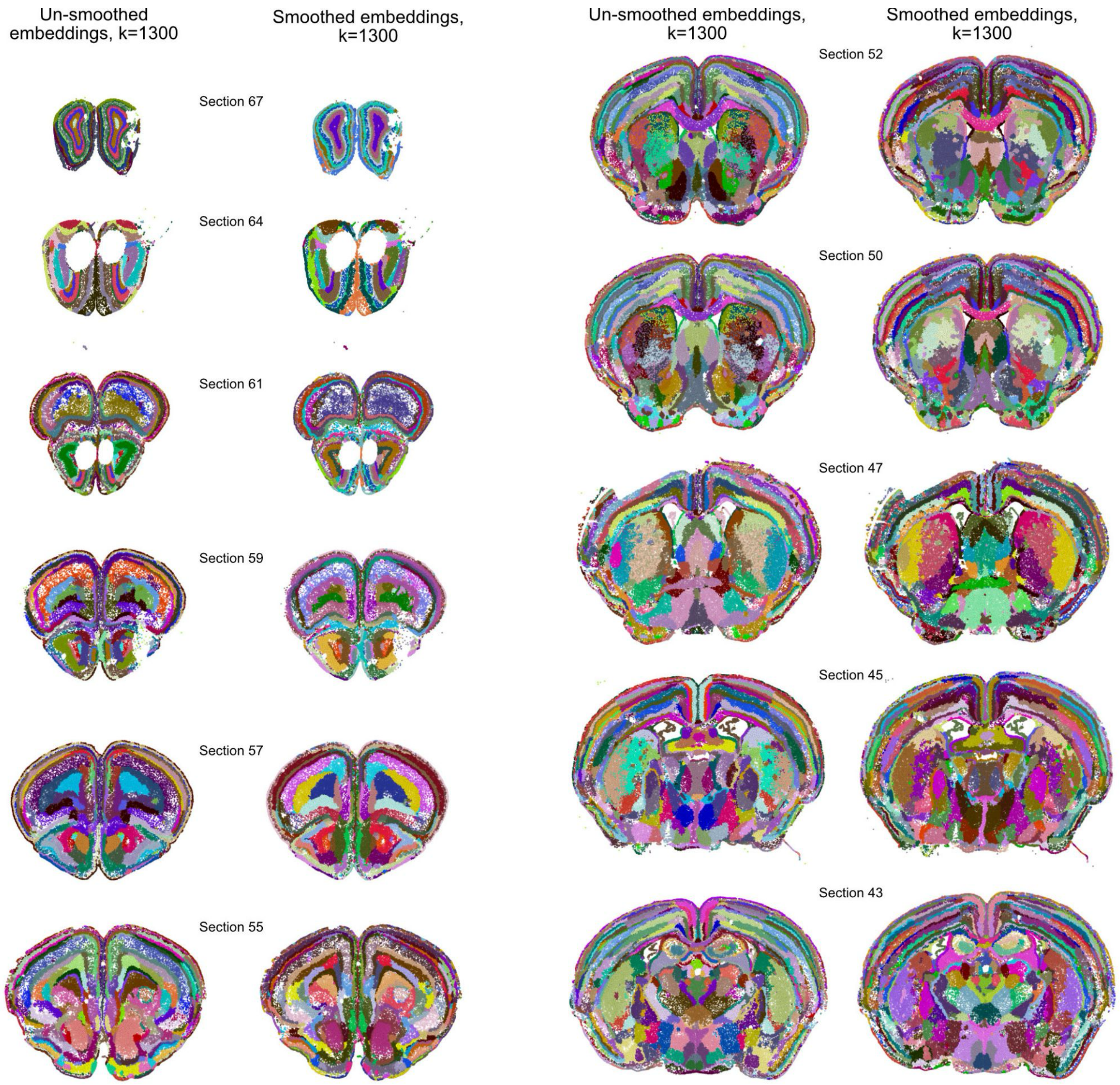

Supplementary Figure 11. Comparison of spatial domains discovered at *k*=1300 domains when using smoothed versus unsmoothed CellTransformer embeddings and focusing on an isolated case of non-uniform spatial domains discussed in **Supplementary Note 1**. Although overall similarity is high, some lamina, particularly in the cortex (sections 45, 43), display slightly altered boundaries. All sections are from the ABC-WMB (Allen 1 dataset).

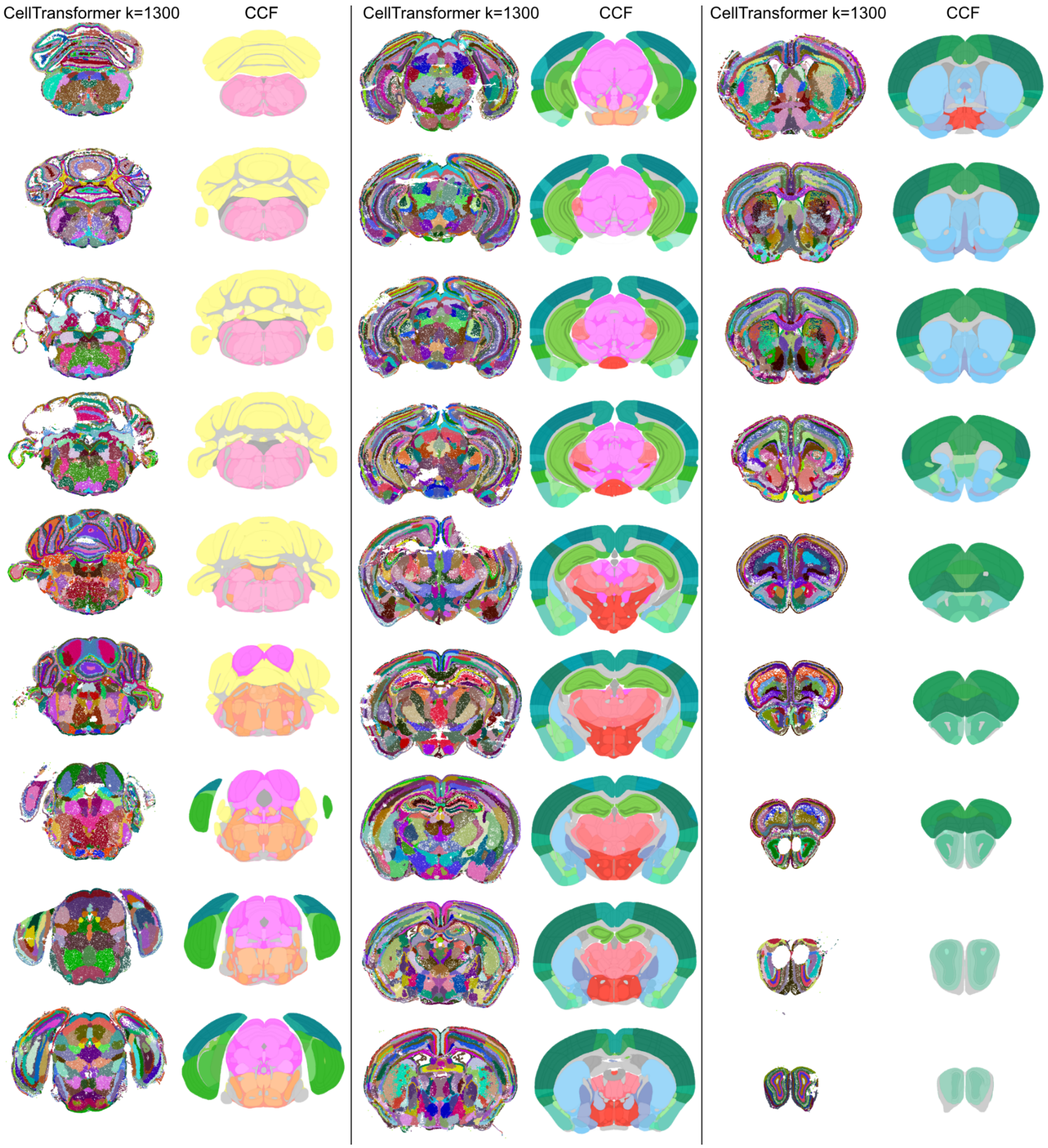

Supplementary Figure 12. CellTransformer spatial domains (left) and the corresponding CCF annotations (right) organized in 3 columns for roughly half of the sections in the Allen 1 dataset[1], approximately every other section. CellTransformer domains were calculated at *k*=1300 clusters without smoothing.

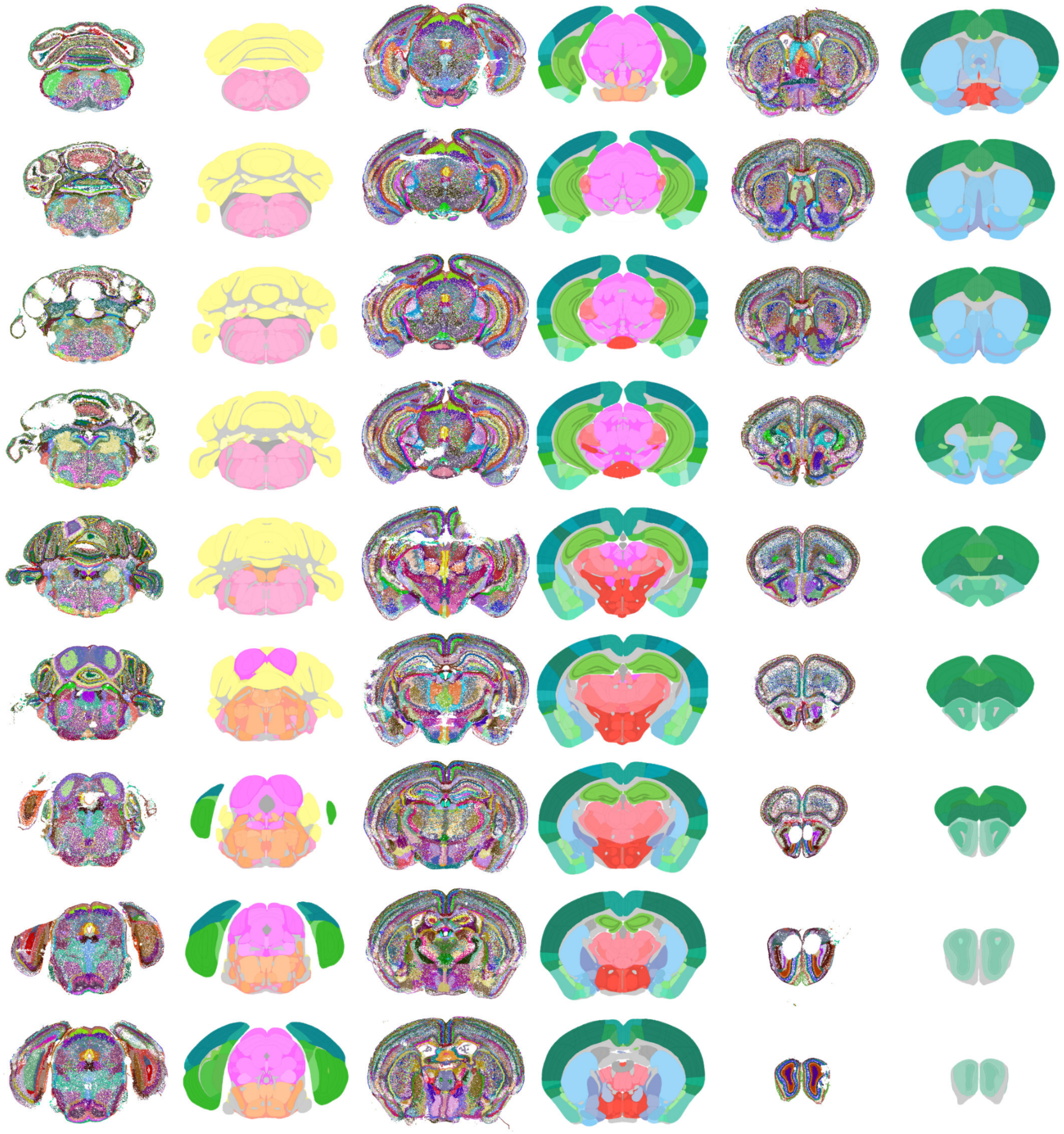

Supplementary Figure 13. CellCharter[4] results (left) and the corresponding CCF annotations (right) organized in 3 columns for roughly half of the sections in the ABC-WMB (Allen 1 dataset)[1], approximately every other section. The color labels for CellCharter correspond to its Gaussian mixture model implementation with *k*=670 clusters.

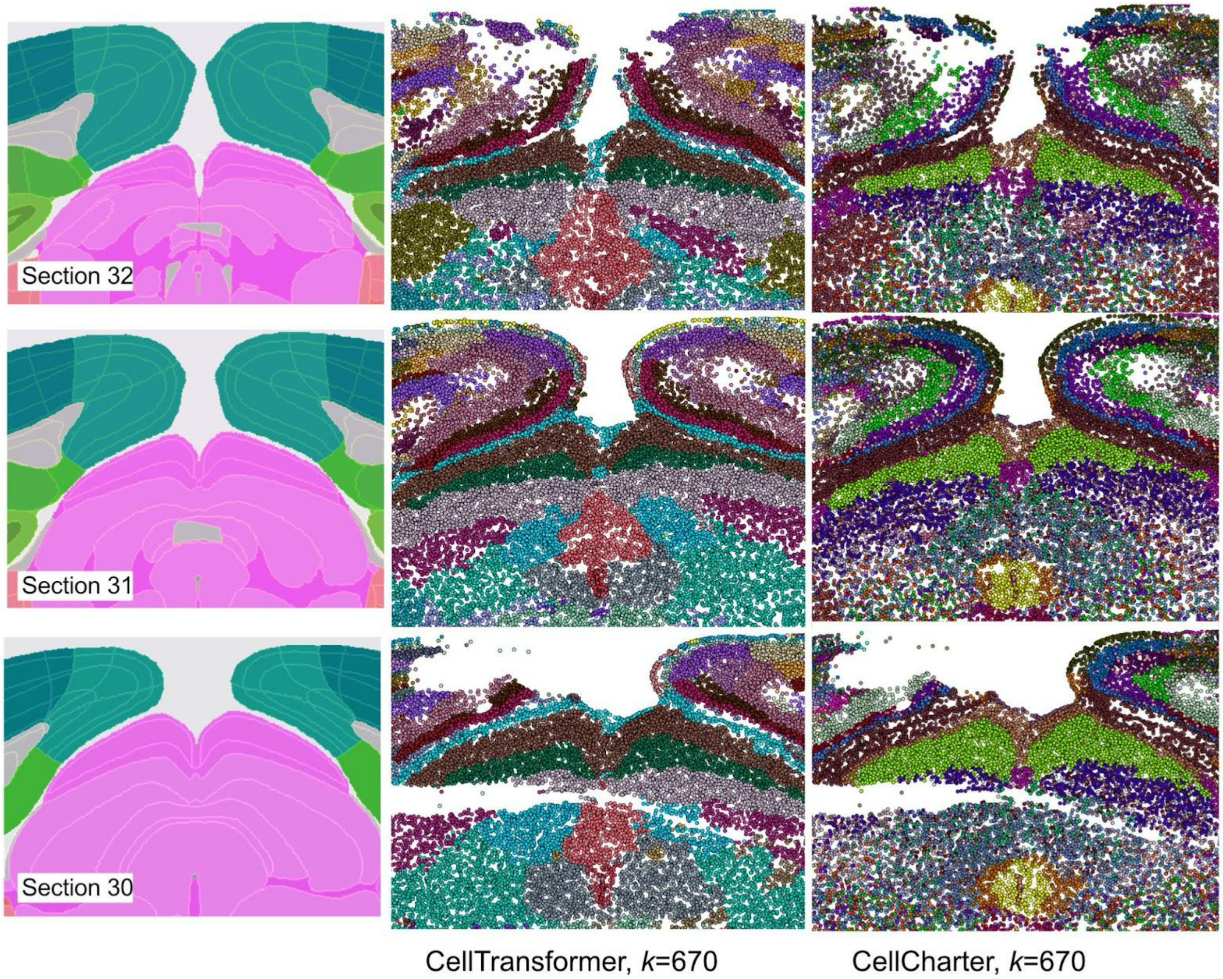

Supplementary Figure 14. Comparison of spatial domains in midbrain for CellTransformer and CellCharter[4] discovered in the Allen 1 dataset[1]. Left column shows approximate CCF registration. Middle column shows CellTransformer domains at *k*=670 and the right column shows CellCharter domains with 670 Gaussians. The general performance in outlining cortical layers is similar, however in the midbrain, even at half the number of clusters, CellCharter loses spatial coherence compared with CellTransformer. For example, CellCharter identifies two layers of superior colliculus, whereas multiple layers are defined by CellTransformer.

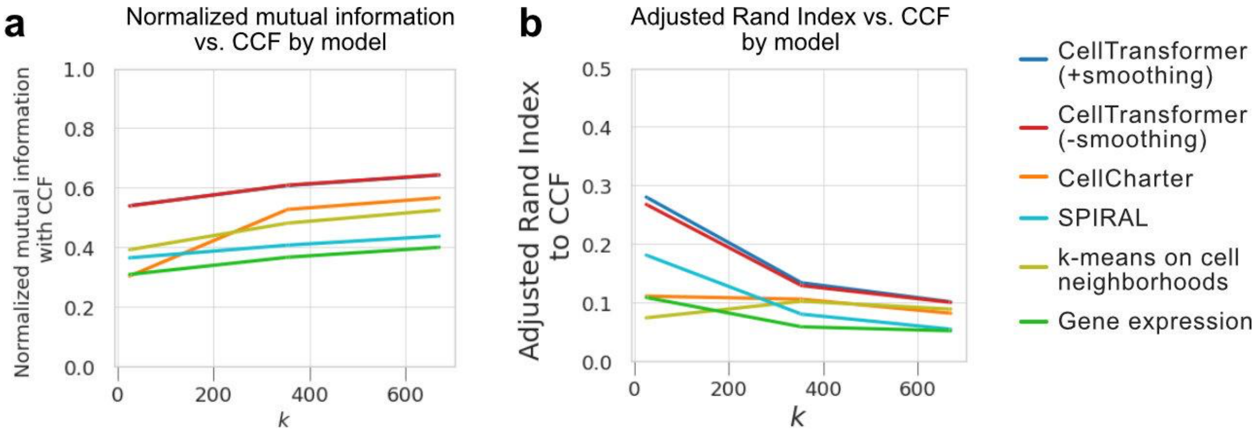

Supplementary Figure 15. Direct quantification of overlap between CCF and data-driven domains in the Allen 1 dataset[1]. (**a.**) Normalized mutual information between data-driven domains and CCF at *k*=25, 354, and 670 domains across different models. (**b.**) Adjusted Rand Index between data-driven domains and CCF at *k*=25, 354, and 670 domains across different models.

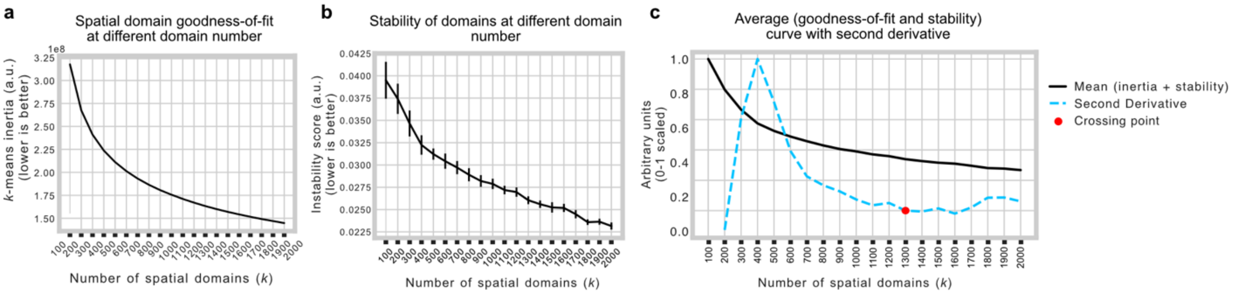

Supplementary Figure 16. Quantification of goodness-of-fit and stability of varying numbers of spatial domains. (**a.**) Inertia (sum of squares errors for each cluster centroid) calculated for different clustering solutions when clustering embeddings generated using CellTransformer on the Allen 1 dataset[1]. Error bars (standard deviation) are calculated but not visible due to scale. (**b.**) Instability scores (see **Methods**) calculated for different clustering solutions using the Allen 1 dataset. Error bars are the standard deviation. (**c.**) Average of inertia and stability curves (black line) and second derivative of the same curve (blue dotted lines). Second derivative crossing point at *k*=1300 shown with a red dot. All comparisons were performed across a range of 100 to 2000 domains in increments of 100.

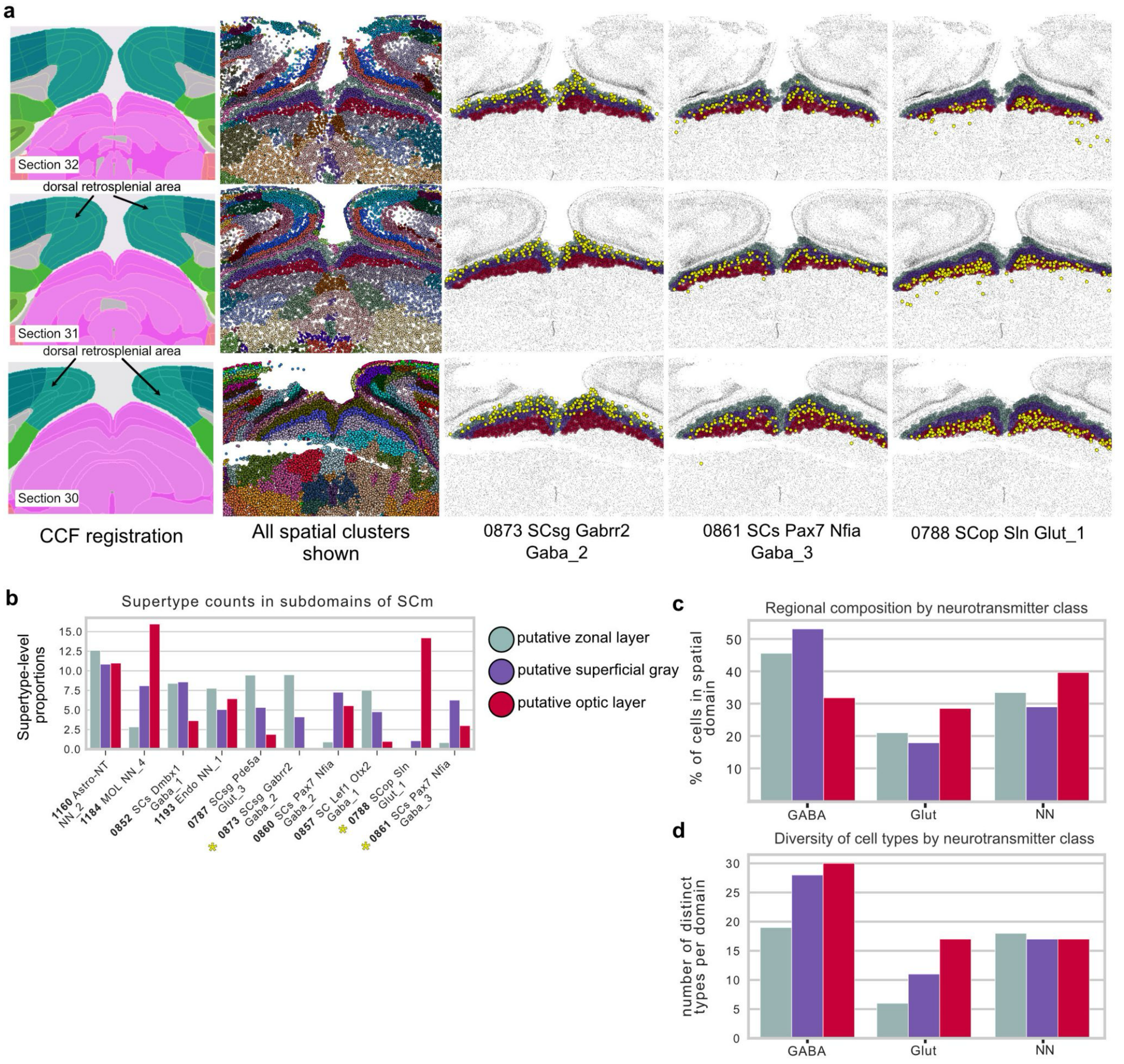

Supplementary Figure 17. Comparison of best-fit spatial domains from CellTransformer with layers of the superior colliculus, sensory-related area. (**a.**) Sequential tissue sections (32, 31, 30, from anterior to posterior). First column: CCF registration and borders of relevant areas. Second column: all cells in the field of view, colored by spatial domain from CellTransformer. Third column: only visualizing cells inside our putative matches for the zonal, superficial gray, and optic layers in the superior colliculus. The 0879 SCsg Pde5a Glut_1 cell type (supertype-level) in yellow. Fourth column: same as third, but visualizing the 0865 SCs Pax7 Nfia Gaba_3 cell type. Fifth column: same as third and fourth, but visualizing the 0882 SCop Sln Glut type. (**b.**) Bar chart of cell type abundance (as a percentage) for the top ten most abundant types across the putative subregions. Cell types visualized in (**a.**) are marked with a yellow asterisk. (**c.**) Bar chart of per-region proportions of GABA-ergic and glutamatergic neurons and non-neuronal types. (**d.**) Bar chart of the number of distinct cell types at supertype level of the ABC-WMB taxonomy per domain.

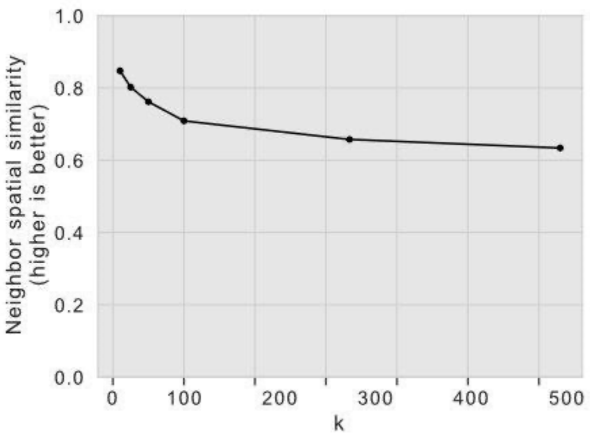

Supplementary Figure 18. Results of quantitative comparison of CCF regions and CellTransformer regions at an equivalent number of regions using the Zhuang 1-4 datasets[5]. Spatial smoothness of spatial clusters as measured using a nearest-neighbors approach, computed by clustering the concatenated latent variables for neighborhoods in the Zhuang lab datasets.

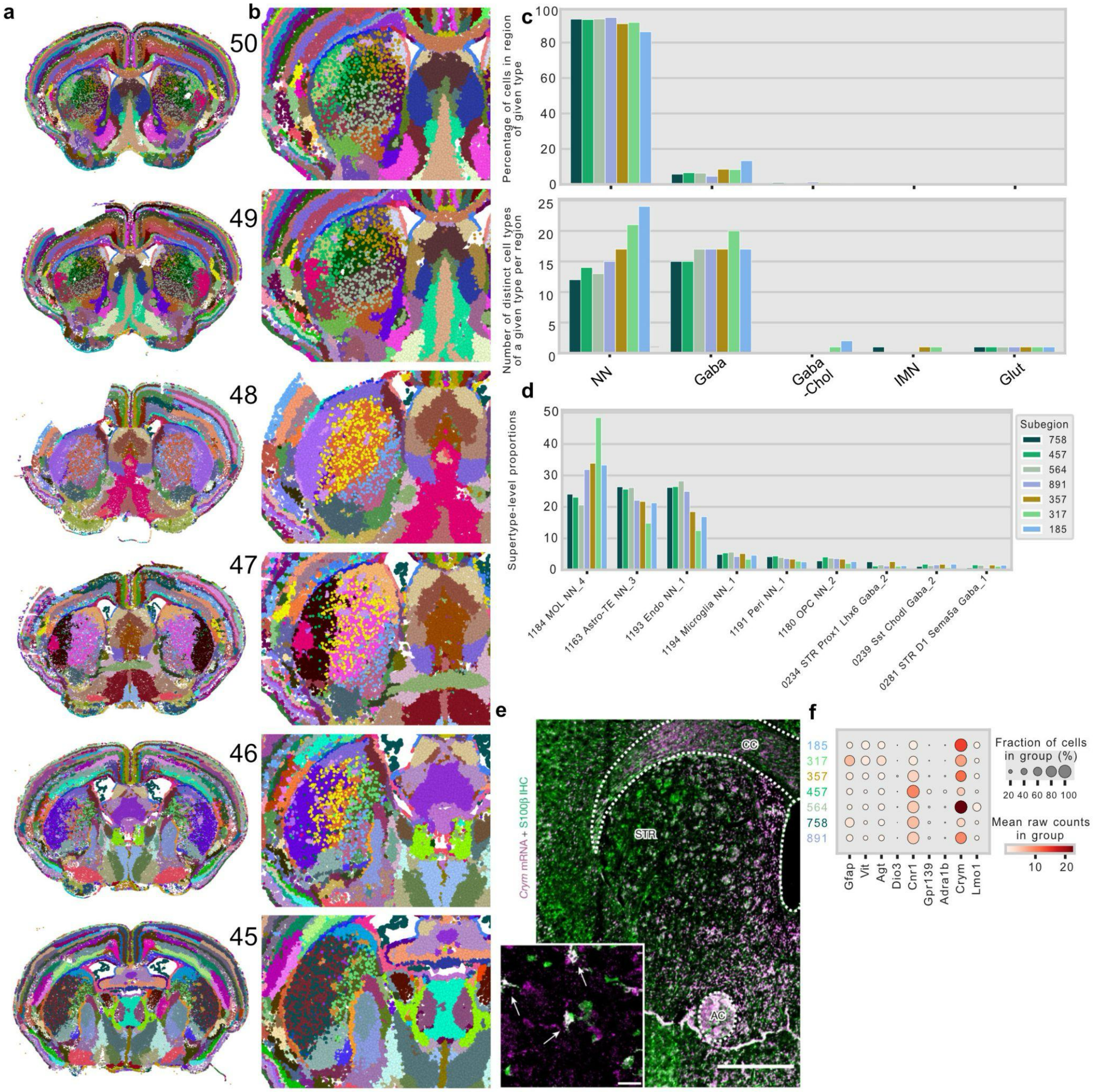

Supplementary Figure 19. Representative images of spatial clustering from CellTransformer models with *k*=1300 identified using the Allen 1 dataset[1]. (**a.**) Sequential tissue sections (50 is most anterior) showing smoothness of spatial domains across and within tissue sections as well as consistent appearance of an irregular spatial pattern inside caudoputamen. (**b.**) Zoom in on the striatum for the same tissue sections. (**c.**) Plots showing percentage of cell types of different neurotransmitter for the non-uniform spatial clusters as well as the distribution of unique cell types of a given neurotransmitter type. (**d.**) Supertype-level counts in putative subpopulations of caudoputamen. (**e.**) Reproduction with permission of results from Ollivier et al. (2024), showing the distribution of *Crym* mRNA and its protein product (S100B), clearly identifying a medial population of *Crym*+ neurons which resembles the spatial pattern observed in clusters 758 and 457 (dorsoventral and Crym^{-}). (**f.**) Dotplot of cell type expression proportions and mean counts per group (raw counts) in identified irregular caudoputamen areas.

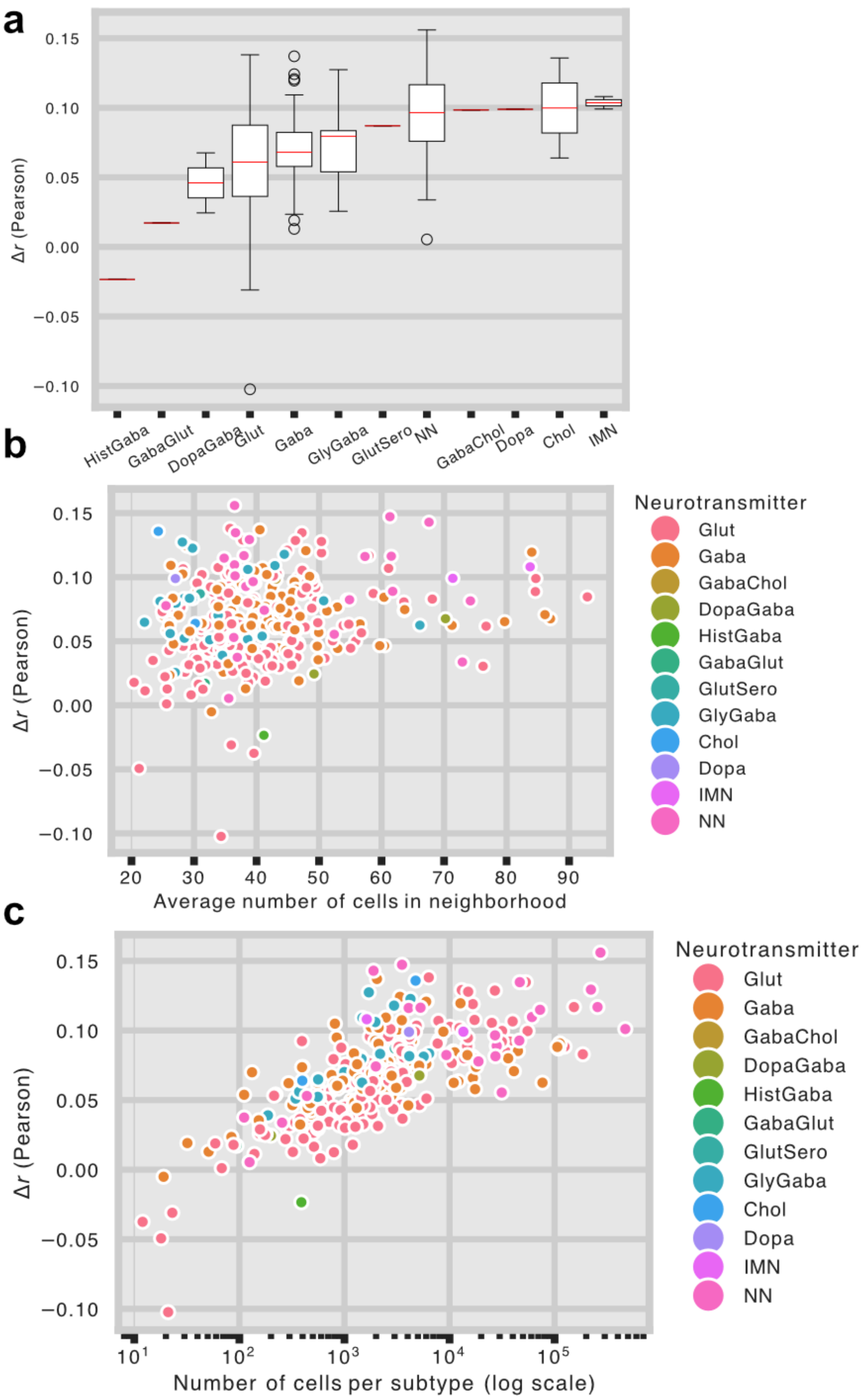

Supplementary Figure 20. Quantification of improved prediction accuracy as a result of CellTransformer's neighborhood-conditioned prediction. Results are over all cells in the Allen 1 dataset[1]. (**a.**) Change in Pearson correlation from per-cell type (at subclass level) average expression. Red lines show medians per distribution. (**b.**) Scatterplot of increase in average Pearson correlation per subclass level cell type against average neighborhood size for reference cells of that type. (**c.**) Scatterplot of increase in average Pearson correlation per subclass level cell type vs the number of cells of that type in log scale.

References

1. Yao, Z. *et al*. A high-resolution transcriptomic and spatial atlas of cell types in the whole mouse brain. *Nature* **624**, 317–332 (2023).
2. Ollivier, M. et al. Crym-positive striatal astrocytes gate perseverative behaviour. Nature 627, 358–366 (2024).
3. Fischer, D. S., Schaar, A. C. & Theis, F. J. Modeling intercellular communication in tissues using spatial graphs of cells. *Nat. Biotechnol.* **41**, 332–336 (2023).
4. Varrone, M., Tavernari, D., Santamaria-Martínez, A., Walsh, L. A. & Ciriello, G. CellCharter reveals spatial cell niches associated with tissue remodeling and cell plasticity. *Nat. Genet.* **56**, 74–84 (2024).
5. Zhang, M. *et al*. Molecularly defined and spatially resolved cell atlas of the whole mouse brain. *Nature* **624**, 343–354 (2023).
